# Effects of Graded Hydroxylated Lecithin Supplementation on Growth Performance, Serum Lipid-Related Indices, Meat Quality, and Tissue-Specific Gene Expression in Jiangnan White Geese

**DOI:** 10.3390/vetsci13080843

**Published:** 2026-08-21

**Authors:** Dexin Zhao, Haoliang Chai, Linfeng Zhou, Ruicheng Han, Weiqi Peng, Li Xu, Hongzhi Wu

**Affiliations:** 1 Tropical Crops Genetic Resources Institute, Chinese Academy of Tropical Agricultural Sciences, Haikou 571101, China; 2 College of Animal Science and Technology, Northeast Agricultural University, Harbin 150030, China

**Keywords:** hydroxylated lecithin, Jiangnan White goose, lipid metabolism, meat quality, growth performance

## Abstract

Conventional soybean lecithin has limited hydrophilicity, which may restrict its efficacy as a feed emulsifier. This study evaluated graded hydroxylated lecithin supplementation in Jiangnan White geese. Six hundred one-day-old females were allocated to five treatments, with six pens of 20 geese each: a basal diet, 50, 100, or 200 mg/kg hydroxylated lecithin, and 100 mg/kg conventional soybean lecithin. The pen served as the experimental unit (*n* = 6). After 64 days, all lecithin-supplemented groups had higher final body weight and average daily gain than the control (*p* < 0.05), whereas 100 and 200 mg/kg hydroxylated lecithin reduced feed conversion ratio (*p* < 0.05). After false-discovery-rate adjustment within prespecified outcome families, robust treatment effects remained for selected serum indices, leg-muscle shear force, muscle-fibre morphology, inosine monophosphate, several flavour-associated amino acids, selected fatty acid outcomes, and tissue-specific expression of the candidate genes (*q* < 0.05). Nominal differences in intramuscular fat, carcass traits, body measurements, and several individual fatty acids did not withstand adjustment and were treated as exploratory. The equal-dose comparison did not show a consistent advantage over conventional soybean lecithin. Overall, hydroxylated lecithin elicited endpoint- and dose-dependent responses, but their commercial relevance and underlying mechanisms require confirmation.

## Simple Summary

Goose meat is a valuable source of dietary protein, but improving feed efficiency while maintaining desirable meat characteristics remains a challenge in commercial goose production. Conventional soybean lecithin has limited water dispersibility, which may constrain its nutritional effects. In a 64-day feeding trial, 600 female Jiangnan White geese were assigned to a basal diet, three diets containing 50, 100, or 200 mg/kg hydroxylated lecithin, or a diet containing 100 mg/kg conventional soybean lecithin. Growth performance, serum lipid-related and antioxidant indices, carcass traits, meat quality, flavour-associated components, fatty acid composition, and the expression of selected lipid metabolism-related genes in the liver, breast muscle, and leg muscle were evaluated. Hydroxylated lecithin supplementation was associated with higher final body weight and average daily gain, while the 100 and 200 mg/kg treatments reduced the feed conversion ratio relative to the basal diet. The treatments also altered selected serum indices, muscle shear force, flavour-associated components, and tissue-specific gene expression. The two lecithin sources produced partly distinct response profiles, but neither source showed a uniform advantage across all measured outcomes. These findings provide evidence for further evaluation of hydroxylated lecithin in goose diets, while the underlying mechanisms require direct functional investigation.

## 1. Introduction

Driven by surging global demand for goose meat, goose production systems are undergoing a transformative shift from extensive free-range rearing to large-scale, intensive, and high-efficiency commercial farming [1,2]. Despite continuous genetic improvement that unlocks greater growth potential, the industry remains hampered by persistent bottlenecks: suboptimal feed efficiency, excessive abdominal fat accretion, and limited capacity to elevate meat quality [3,4]. Modern consumers no longer prioritise carcass yield alone; instead, they place greater emphasis on tenderness, juiciness, distinctive flavor profiles, and the nutritional value of goose meat [5]. Reconciling superior productive performance with improved meat quality has therefore emerged as a central objective in waterfowl nutrition research [6]. Establishing precision nutrition strategies to modulate whole-body lipid metabolism and achieve a balanced trade-off between growth rate, adipose partitioning, and meat quality represents a critical scientific frontier underpinning high-quality sustainable goose production [7].

Lipids serve not only as the primary energy substrate for animal growth and development but also constitute integral structural components of cell membranes [8]. They participate in a broad spectrum of physiological processes, including the absorption of fat-soluble vitamins, cellular signal transduction, inflammatory modulation, and the maintenance of cellular redox homeostasis [9,10]. Accumulating evidence indicates that targeted manipulation of lipid metabolism enhances dietary energy utilisation, remodels adipose tissue distribution, promotes intramuscular fat deposition, and modulates polyunsaturated fatty acid and flavour-active compound profiles, thereby shaping the sensory quality of livestock and poultry meat, though changes in PUFA fractions and n-6/n-3 ratio require cautious nutritional interpretation [11,12,13]. This regulatory framework carries particular relevance for waterfowl such as geese, which exhibit inherently robust lipid turnover and unique adipose partitioning patterns [14]. Refined lipid nutritional strategies are therefore indispensable for balancing productive outputs and meat quality in goose production. As a natural source of phospholipids, lecithin performs multiple biological functions, including promoting lipid emulsification, improving lipid digestibility, and regulating systemic lipid metabolism, making it a widely used feed additive in livestock and poultry production [15,16]. Numerous feeding trials have validated that dietary lecithin supplementation boosts lipid emulsification, stimulates lipase activity, and accelerates lipid transport, yielding moderate improvements in animal growth, reduced circulating lipid concentrations, and enhanced meat quality [17,18]. Additionally, lecithin acts as a major choline donor to support lipoprotein biogenesis and hepatic lipid efflux, exerting protective effects on whole-body lipid homeostasis [19]. Against this backdrop, structural modification of lecithin to amplify its nutritional functionality has evolved into a vibrant research niche within animal nutrition science. Hydroxylated lecithin is a modified phospholipid characterised by an increase in the hydrophilic–lipophilic balance (HLB) value from 3–4 to 10–12, thereby improving water dispersibility, emulsion stability, and antioxidant capacity. Geese exhibit pronounced hepatic lipid deposition, tissue-specific lipid partitioning, and PUFA-rich skeletal muscle that responds readily to dietary lipid manipulation, making them an ideal model for investigating lipid nutritional regulation. The selected supplementation levels (50, 100, and 200 mg/kg) were determined based on our preliminary study [20], the manufacturer’s recommended inclusion range (100–300 mg/kg), and the need to establish a dose–response relationship using approximately two-fold dose increments.

Notwithstanding these documented benefits, conventional soybean lecithin delivers inconsistent performance under commercial production conditions [21,22]. Its limited hydrophilic moieties compromise water solubility and dispersibility, favouring the formation of large lipid aggregates within the digestive tract and thereby curtailing emulsification efficiency and in vivo bioavailability [21]. Furthermore, its ability to regulate lipid partitioning among tissues remains limited, making it difficult to simultaneously reduce abdominal fat deposition, promote intramuscular lipid accumulation, and improve both growth performance and meat quality [22]. These drawbacks are exacerbated in waterfowl with inherently hyperactive lipid metabolism, hindering broader application of unmodified soybean lecithin in high-yield goose farming. Hydroxylation has recently emerged as a promising chemical modification strategy to potentiate phospholipid bioactivity. Incorporation of hydroxyl functional groups rebalances the hydrophilic–lipophilic character of phospholipid molecules, markedly improving water dispersibility and emulsion stability [23]. This structural alteration also elevates lipid digestibility, promotes lipoprotein assembly, and facilitates transmembrane fatty acid transport, ultimately reshaping systemic lipid trafficking and tissue-specific fat deposition patterns [24]. Our previous study showed that dietary hydroxylated lecithin affected growth performance and several physiological traits in Jiangnan White goslings during the early rearing period from 1 to 32 days. However, that study did not determine whether these responses persisted throughout the commercial production cycle, nor did it evaluate muscle fatty acid composition, flavour-associated amino acids, or tissue-specific expression of lipid metabolism-related genes. Evidence directly comparing hydroxylated lecithin with conventional soybean lecithin at the same inclusion level also remains limited in geese. Therefore, whether hydroxylation is associated with broader physiological responses and distinct hepatic and muscular transcriptional patterns has not been fully established. The present study used a completely independent population of geese, newly collected biological samples, and an independent dataset. Its main advances were the extension of the feeding period to the complete 64-day commercial production cycle, the inclusion of an equal-dose conventional soybean lecithin reference group, and the integrated evaluation of growth performance, serum lipid-related and antioxidant indices, carcass traits, meat quality, flavour-associated components, fatty acid composition, and selected gene-expression responses in the liver, breast muscle, and leg muscle.

Accordingly, we hypothesised that the improved amphiphilic properties of hydroxylated lecithin would be associated with differences in lipid-related physiological responses and tissue-specific gene expression, accompanied by changes in growth performance and meat-quality characteristics. To test this hypothesis, female Jiangnan White geese were fed graded levels of hydroxylated lecithin, with an equal inclusion level of conventional soybean lecithin serving as the reference treatment. The study evaluated growth performance, serum lipid-related and antioxidant indices, carcass traits, muscle physicochemical characteristics, flavour-associated components, fatty acid composition, and the expression of selected lipid metabolism-related genes in the liver, breast muscle, and leg muscle.

## 2. Materials and Methods

All animal husbandry and experimental procedures involved in this study were approved by the Institutional Animal Care and Use Committee of the Chinese Academy of Tropical Agricultural Sciences (approval number: CATAS20250601-IV). The entire experimental workflow was conducted in strict compliance with the relevant laws and regulations of the local jurisdiction, as well as the institutional requirements.

### 2.1. Animals, Diets, Design, and Management

A total of 600 healthy one-day-old female Jiangnan White geese with homogeneous initial body weight were randomly allocated into five dietary treatments, with six replicate pens per treatment and 20 female geese housed in each replicate pen. Only female geese were used to minimise sex-related variation in growth rate, carcass composition, and lipid deposition, thereby maintaining a sex-uniform experimental population. The feeding trial lasted 64 days, and all biological samples were collected at the end of the experiment. The dietary treatments consisted of a control group (CG) fed the basal diet without lecithin supplementation, three hydroxylated lecithin groups supplemented with 50 (HLG50), 100 (HLG100), or 200 mg/kg (HLG200), and a soybean lecithin reference group supplemented with 100 mg/kg conventional soybean lecithin (LG100). Hydroxylated lecithin and conventional soybean lecithin were supplied by Dalian Huanong Beans Technology Development Co., Ltd (Pulandian New District, Dalian City, Liaoning Province, China).

The detailed specifications of the two lecithin products, as provided by the manufacturer, are summarised below. Hydroxylated lecithin (CAS No. 8029-76-3) is a modified soybean lecithin produced by treating natural phospholipids with hydrogen peroxide in the presence of lactic acid or acetic acid, followed by neutralisation with sodium hydroxide. The hydroxylation process reduces the iodine value from 90–100 to 78–90 (approximately 10% reduction), and increases the hydroxyl value from 145–160 to 170–185. The product is a light yellow powder/granule without carriers, with an HLB value of 10–12 (compared to 3–4 for non-hydroxylated lecithin), indicating substantially improved water dispersibility.

The major compositional parameters of the hydroxylated lecithin product were: acetone-insoluble matter (phospholipids) 58–60%, moisture 0.8–1.2%, acid value 30–35, peroxide value 2–4, and sulfate ash 0.1–0.5%. The phospholipid fraction consists chiefly of phosphatidyl choline, phosphatidyl ethanolamine, and phosphatidyl inositol, along with other minor phospholipids and glycolipids. The product contains no carriers or diluents. Regarding residual reagents from the hydroxylation process, the manufacturer confirmed that hydrogen peroxide and organic acid (lactic or acetic acid) are consumed during the reaction, and the final product is neutralised with sodium hydroxide; residual peroxide levels are monitored by the peroxide value (2–4), and residual organic acid is controlled within specification limits. The product batch number used in this study was HS-20240315 (hydroxylated lecithin) and SL-20240318 (soybean lecithin).

The conventional soybean lecithin product (non-hydroxylated) had the following specifications: acetone-insoluble matter (phospholipids) 62–64%, moisture 0.2–0.4%, acid value 20–25, iodine value 90–100, peroxide value 0–1.0, and sulfate ash 0.1–0.5%. Its HLB value is 3–4, reflecting lower hydrophilicity compared to the hydroxylated counterpart.

To verify the thermal stability of both additives during feed pelleting (which involves conditioning temperatures of 75–80 °C), the manufacturer provided stability data indicating that the phospholipid content remains ≥ 95% of the initial value after exposure to 80 °C for 30 min, with no significant degradation of active components. This is consistent with the known thermal stability of hydroxylated lecithin derivatives. The basal diet was formulated to meet the nutritional requirements of meat geese in accordance with the recommendations of the *Nutrient Requirements of Poultry*, 10th Revised Edition, published by the National Academies of Sciences, Engineering, and Medicine (2026), and all experimental feeds were processed into pellet form to reduce feed waste and selective feeding of geese. To verify the actual lecithin levels in the finished pelleted diets after feed manufacture, representative feed samples (three sub-samples per treatment) were collected immediately after pelleting and analysed for acetone-insoluble matter (AI), the standard quality-control index for lecithin products, according to the manufacturer’s validated analytical protocol. Because AI in complete diets represents the total acetone-insoluble fraction derived from both endogenous feed ingredients and supplemented lecithin products, the analysed values represent the measured total AI content of the finished diets rather than theoretical phospholipid concentrations calculated from the inclusion rates of the additives. The analysed total AI contents of the finished pelleted diets were 0.12 ± 0.01% for CG (basal diet), 0.17 ± 0.02% for HLG50, 0.22 ± 0.02% for HLG100, 0.32 ± 0.03% for HLG200, and 0.21 ± 0.02% for LG100. These values represent analytical determinations of the finished pelleted diets and were used solely to verify the consistency of feed manufacture and additive incorporation.

The detailed dietary composition and nutrient levels are presented in Table 1. Mortality and culling were recorded daily throughout the 64-day trial. Birds that died or were culled due to injury or illness were excluded from final performance calculations. To adjust feed intake and feed conversion ratio (FCR) for mortality, total feed consumption was corrected by subtracting the estimated feed consumed by birds before death or culling. Specifically, feed intake was corrected using the method of excluding feed consumed by dead birds based on the proportion of dead bird weight to total pen weight, and mortality-corrected FCR was calculated as corrected feed intake/(final live weight of surviving birds + weight of dead birds − initial weight of all birds). No birds were excluded as outliers during the trial.

Throughout the 64-day trial, all geese were housed on fully slatted plastic floors under standardised environmental conditions. Natural light was supplemented with artificial lighting from 05:00 to 21:00 daily, while ambient temperature and relative humidity were maintained at 18–26 °C and 55–70%, respectively. Feed and fresh drinking water were provided ad libitum via hanging feeders and automatic nipple drinkers. No antibiotics, growth promoters, or additional lipid additives were included in any experimental diet. Residual feed in each pen was weighed at 08:00 each morning to determine daily feed intake. Mortality, culling, and health status were recorded daily throughout the experiment. Before sampling, all geese were fasted for 12 h to minimise the influence of gastrointestinal contents on subsequent measurements.

### 2.2. Growth Performance and Body Measurements

Body weight was recorded on days 1 and 64. At placement, each goose was weighed individually using an electronic balance (VS-34, Rice Lake Weighing Systems, USA), and the individual values were used to calculate the initial mean body weight of each pen. At the end of the 64-day feeding period, all surviving geese were weighed after a 12 h feed withdrawal using the same balance. Individual body weights were averaged within each pen to obtain the final pen-level body weight. Therefore, body-weight measurements were conducted twice during the experiment, on days 1 and 64.

Feed offered and residual feed were weighed daily at the pen level using an electronic balance (PX124ZH/E, OHAUS Corporation, Parsippany, NJ, USA). Average daily gain was calculated as the difference between final and initial mean body weight divided by 64 days. Average daily feed intake was calculated from mortality-corrected cumulative feed consumption, feeding duration, and the number of geese in each pen. Feed conversion ratio was calculated as average daily feed intake divided by average daily gain.

All body measurements were obtained from live geese in a calm state. Semi-submerged length was measured from the tip of the beak to the base of the tail, whereas body oblique length was measured from the shoulder joint to the hip joint. Keel length, breast depth, breast width, shank length, shank circumference, and pelvic width were measured according to standard morphometric procedures. All measurements were recorded to the nearest 0.1 cm.

### 2.3. Sample Selection, Blood Collection, Slaughter, and Tissue Sampling

At the end of the 64-day feeding period, two healthy female geese with body weights closest to the mean body weight of each pen were selected after a 12 h feed withdrawal. The two sampled geese originated from the same feeding experiment and were used for all subsequent blood, carcass, meat-quality, biochemical, and gene-expression measurements. Before slaughter, each selected goose was individually weighed and placed in a standardised position for body measurements. All measurements were obtained by the same trained technician using a flexible measuring tape and digital callipers.

Approximately 8 mL of blood was collected from the wing vein into sterile vacuum tubes without anticoagulant. Samples were allowed to clot at room temperature for 30 min and were centrifuged at 3500× *g* for 10 min at 4 °C. The resulting serum was divided into aliquots and stored at −80 °C until analysis.

Following blood collection, the geese were electrically stunned using a handheld stunner (E-500, Euthanex Corporation, Pittsburgh, PA, USA) at 110 V, 50 Hz, and 0.5 A for 3 s and immediately exsanguinated through the jugular vein. Carcass, semi-eviscerated, and eviscerated weights were recorded sequentially. Bilateral breast and leg muscles and abdominal fat were subsequently dissected and weighed. The heart, liver, spleen, glandular stomach, and gizzard were removed, cleared of adhering fat and connective tissue, and weighed using an electronic balance with a precision of 0.01 g.

Liver samples were collected from a standardised site in the left hepatic lobe, while breast- and leg-muscle samples were obtained from standardised anatomical sites on the right side of each bird. Approximately 2 g of each tissue was wrapped in prelabelled aluminium foil, frozen in liquid nitrogen, and stored at −80 °C for fatty acid and amino acid analyses. An additional 10 g of fresh muscle was stored at 4 °C for pH, shear-force, and muscle-fibre measurements. Approximately 0.5 g of liver, breast muscle, and leg muscle was frozen in liquid nitrogen and stored at −80 °C for RNA extraction. Tissue collection was completed within 10–15 min after exsanguination.

Measurements obtained from the two geese sampled from the same pen were averaged to generate a single pen-level value. Therefore, the pen was used as the experimental unit for all statistical analyses, with six biological replicates per treatment.

### 2.4. Determination of Carcass Traits and Organ Indices

After electrical stunning and exsanguination, each sampled female goose was weighed for live weight before slaughter, followed by hair removal and carcass weighing to calculate slaughter rate (SR); after removing trachea, crop and intestinal tract except heart, liver and gizzard, semi-eviscerated weight was measured, and all internal organs were stripped to record eviscerated weight for calculating semi-eviscerated rate (semi-ER) and eviscerated rate (ER). Bilateral breast muscle and leg muscle were completely dissected and weighed to calculate breast muscle rate (BR) and leg muscle rate relative to live weight; separated abdominal fat was weighed to calculate belly fat rate (BFR). Heart, liver, spleen, glandular stomach, and gizzard were stripped of surface fat and fascia and weighed with a 0.01 g precision electronic balance; each organ index was expressed as the percentage of organ weight relative to live weight, and all weighing operations were finished within 10 min after dissection to avoid tissue water loss causing weight deviation.

### 2.5. Laboratory Analyses

#### 2.5.1. Serum Lipid Metabolism-Related Biochemical Indices

Serum samples were removed from the −80 °C freezer and thawed on ice. After gentle vortexing, samples showing hemolysis, visible lipemia, or precipitation were excluded from subsequent analysis. Serum alanine aminotransferase (ALT), lipoprotein lipase (LPL), superoxide dismutase (SOD), glutathione peroxidase (GSH-Px), and malate dehydrogenase (MDH) activities were determined using commercial colorimetric kits (Nanjing Jiancheng Bioengineering Institute, Nanjing, China) according to the manufacturer’s instructions. Absorbance was measured using a Hitachi 7100 automated biochemical analyzer (Hitachi 7100 automated biochemical analyzer, Hitachi High-Technologies Corporation, Tokyo, Japan), and enzyme activities were calculated according to the kit protocols. Serum concentrations of adiponectin (ADPN), leptin (LEP), glucagon (GLC), insulin (INS), thyroxine (T4), triiodothyronine (T3), and thyrotropin-releasing hormone (TRH) were measured using commercially available ELISA kits (Shanghai Enzyme-linked Biotechnology, Shanghai, China). All samples were analysed in duplicate, and only standard curves with an R^2^ value > 0.99 were accepted. Conventional serum lipid and antioxidant indicators high-density lipoprotein (HDL), low-density lipoprotein (LDL), apolipoprotein A1 (Apo-A1), apolipoprotein B (Apo-B), total cholesterol (TC), triglycerides (TG), glucose (GLU), free fatty acid (FFA), malondialdehyde (MDA) and total glutathione (T-GSH) concentrations were determined using commercial assay kits according to the manufacturer’s instructions. All analyses were completed within 3 h after thawing to minimise sample degradation.

#### 2.5.2. Muscle pH, Shear Force, and Muscle-Fibre Morphology

Fresh breast and leg muscle samples collected within 2 h postmortem without water washing were used for physical quality measurements. A Testo 20 portable pH meter (Testo SE & Co. KGaA, Titisee-Neustadt, Germany) was inserted approximately 2 cm into the central region of each muscle to determine pH at 15 min postmortem. Shear force was determined using the Warner–Bratzler method. Muscle samples measuring 1 × 1 × 3 cm were cut parallel to the muscle fibres, sealed in plastic film, heated in a water bath at 75 °C for 30 min, cooled to room temperature, and analysed using a TA-XT Plus texture analyser equipped with a Warner–Bratzler shear blade. Three technical measurements were obtained for each sample and averaged before statistical analysis.

For muscle-fibre morphology, tissue blocks measuring approximately 0.8 × 0.8 × 0.5 cm were fixed in 4% paraformaldehyde for 24 h, dehydrated through a graded ethanol series, cleared in xylene, embedded in paraffin, and sectioned at 5 μm for haematoxylin and eosin staining. Five non-overlapping fields were randomly photographed under an optical microscope using a 10× objective. ImageJ software (version 1.54r, National Institutes of Health, Bethesda, MD, USA) was used to determine muscle-fibre diameter and fibre density per square millimetre.

#### 2.5.3. Muscle Flavour Precursor Substances

Frozen breast and leg muscle samples were thawed on ice, and visible fat and connective tissue were removed. Approximately 1 g of tissue was homogenised in ice-cold phosphate buffer and centrifuged at 4000× *g* for 15 min at 4 °C. The supernatant was collected for determination of intramuscular fat (IMF), inosinic acid (INO), triglycerides (TG), and phospholipids (PL). IMF was extracted by petroleum ether reflux method and quantified by weighing after solvent volatilization; INO, TG and PL all adopted tissue colorimetric kits; corresponding standard substances were used to prepare standard curves for content calculation, and two technical replicates were set for each sample to reduce operational error.

#### 2.5.4. Muscle Fatty Acid Composition

Approximately 2 g of frozen breast or leg muscle was minced, and total lipids were extracted twice with chloroform:methanol (2:1, *v*/*v*). The combined extracts were evaporated under nitrogen before methyl ester preparation. Sequential saponification in a 70 °C water bath and boron trifluoride methyl esterification were conducted; n-hexane was used to extract fatty acid methyl esters, and the extract was filtered through a 0.22 μm organic membrane before machine injection. An Agilent 7890A GC-MS (Agilent Technologies, Inc., Santa Clara, CA, USA) equipped with a DB-FAT column (60 m × 0.25 mm × 0.20 μm) was used; helium carrier gas was at 1.0 mL/min. Oven programme: 140 °C (5 min), 4 °C/min to 240 °C (15 min); injector 250 °C, detector 280 °C. Fatty acids were identified by Supelco 37 standards and expressed as g/100 g total FA. Total saturated fatty acids (SFA) were calculated as the sum of C14:0, C16:0, C18:0, C20:0, C22:0 and C24:0; total monounsaturated fatty acids (MUFA) as the sum of C16:1, C18:1n9c, C20:1 and C24:1; total polyunsaturated fatty acids (PUFA) as the sum of C18:2n6c, C18:3n3, C20:2, C20:3, C20:4n6, C20:5 and C22:6n3; total n-3 PUFA as the sum of C18:3n3, C20:5 and C22:6n3; and total n-6 PUFA as the sum of C18:2n6c, C20:3 and C20:4n6. The n-6/n-3 ratio was subsequently calculated.

#### 2.5.5. Muscle Amino Acid Composition

Approximately 1 g of defatted breast or leg muscle homogenate was hydrolyzed with 6 mol/L hydrochloric acid in sealed tubes under nitrogen at 110 °C for 22 h. After hydrolysis, samples were cooled, neutralised, adjusted to a constant volume, filtered through a 0.22 μm membrane, and derivatized with phenyl isothiocyanate prior to analysis. Samples were separated and quantified by Agilent 1260 high-performance liquid chromatography (HPLC) with a C18 reversed-phase column; mobile phase A was sodium acetate buffer and mobile phase B was acetonitrile with gradient elution, ultraviolet detection wavelength set at 254 nm; an amino acid mixed standard was used for quantitative calibration. The amino acids selected for analysis included aspartic acid (Asp), serine (Ser), glutamic acid (Glu), glycine (Gly), alanine (Ala), lysine (Lys), histidine (His), and arginine (Arg). These amino acids were selected because they have been reported to contribute to meat taste or to serve as precursors for flavour formation during heating. In particular, Asp and Glu are associated with umami taste, whereas Ser, Gly, and Ala contribute mainly to sweet taste; Lys, His, and Arg may contribute to taste complexity or participate in thermally induced flavour-forming reactions. Because acid hydrolysis quantifies total hydrolysable amino acids rather than the free amino-acid fraction, the results were interpreted as the abundance of flavour-associated precursor amino acids and not as a direct measurement of taste intensity [25,26,27,28].

#### 2.5.6. Lipid Metabolism-Related Gene Expression Quantification

Approximately 0.3 g of each frozen tissue, including liver, breast muscle, and leg muscle, was separately ground in a mortar precooled with liquid nitrogen. Total RNA was extracted using TRIzol reagent (Invitrogen, Carlsbad, CA, USA) according to the manufacturer’s instructions, and all procedures were performed on ice to minimise RNA degradation. RNA concentration and purity were determined using a NanoDrop 2000 spectrophotometer (Thermo Fisher Scientific, Wilmington, DE, USA). Samples with an A260/A280 ratio between 1.8 and 2.1 were considered acceptable. RNA integrity was assessed by 1% agarose gel electrophoresis before reverse transcription. First-strand cDNA was synthesised using the PrimeScript RT Reagent Kit (Takara Bio Inc., Kusatsu, Shiga, Japan) according to the manufacturer’s protocol. Each 20 μL reverse-transcription reaction contained 500 ng of total RNA, 4 μL of 5× PrimeScript RT Master Mix, and RNase-free water. The reaction was performed at 37 °C for 15 min, followed by 85 °C for 5 s, and the resulting cDNA was stored at −20 °C until analysis.

The target genes were selected as a hypothesis-driven candidate panel based on published transcriptomic evidence [29] and their established roles in complementary aspects of lipid metabolism. The panel included: *stearoyl-CoA desaturase* (*SCD*) and *ELOVL fatty acid elongase 6* (*Elovl6*), representing fatty acid desaturation and elongation, respectively; *acyl-CoA synthetase long-chain family member 1* (*ACSL1*), which participates in long-chain fatty acid activation and subsequent metabolic utilisation; *hydroxysteroid 17-beta dehy-drogenase 7* (*HSD17B7*), which is involved in sterol biosynthesis; *high-density lipopro-tein-binding protein* (*HDLBP*), which is associated with lipoprotein-related lipid handling; *coenzyme Q9* (*COQ9*), which contributes to mitochondrial coenzyme Q function; *cytochrome P450 2C45* (*CYP2C45*), which is associated with oxidative lipid metabolism; and *fibronectin 1* (*FN1*), which is related to extracellular-matrix and tissue remodelling. Together, these genes were used to characterise tissue-specific transcriptional responses in the liver, breast muscle, and leg muscle rather than to represent a complete lipid–metabolism pathway. *Glyceralde-hyde-3-phosphate dehydrogenase* (*GAPDH*) was used as the reference gene. Because reference-gene stability was not formally evaluated across all three tissues and treatments and no second reference gene was available for geometric normalisation, the qPCR data are interpreted as supportive, hypothesis-generating evidence rather than definitive pathway validation. Primer sequences, amplicon sizes, and corresponding GenBank accession numbers are listed in Table 2.

Quantitative real-time PCR was performed using a Roche LightCycler 480 system (Roche Diagnostics, Rotkreuz, Switzerland) with a SYBR Green detection kit. Each 20 μL reaction contained 10 μL of SYBR Green Master Mix, 0.4 μL each of the forward and reverse primers, 2 μL of diluted cDNA template, and 7.2 μL of RNase-free water. The amplification programme consisted of an initial denaturation at 95 °C for 30 s, followed by 40 cycles at 95 °C for 5 s and 60 °C for 30 s. Melting-curve analysis was subsequently performed to assess amplification specificity. Each biological sample was analysed in technical triplicate, and the mean Ct value of the three technical reactions was used for subsequent calculations. Relative mRNA expression was normalised to GAPDH and calculated using the 2^−ΔΔCt^ method, with the CG used as the calibrator.

### 2.6. Statistical Analysis

Growth performance data were analysed using the pen as the experimental unit (six replicate pens per treatment). For endpoint laboratory measurements, including serum biochemical indices, meat quality traits, fatty acid composition, amino acid composition, carcass characteristics, organ indices, and gene expression, two representative geese were sampled from each pen. Duplicate ELISA and HPLC measurements and triplicate qPCR reactions represented technical replicates and were first averaged to obtain a single value for each sampled bird. Subsequently, the values obtained from the two birds sampled within the same pen were averaged to generate one representative value for that pen. Therefore, the pen was regarded as the biological replicate and experimental unit for all statistical analyses (*n* = 6 pens per treatment). Prior to statistical analysis, all datasets were tested for normality using the Shapiro–Wilk test and for homogeneity of variance using Levene’s test. As all variables satisfied these assumptions, no data transformation or non-parametric analysis was required. Data were analysed by one-way analysis of variance (ANOVA) using SPSS software (Version 26.0; IBM Corp., Armonk, NY, USA). To address multiplicity across the large endpoint panel, omnibus ANOVA *p* values were additionally adjusted using the Benjamini–Hochberg procedure within seven prespecified biological families: growth performance, body measurements, serum indices, carcass and organ traits, physical meat quality and muscle morphology, flavour-associated metabolites and amino acids, and fatty acid composition. The eight gene-expression endpoints were adjusted separately within each tissue. Adjusted values are reported as false-discovery-rate q values. An outcome was considered robust to multiplicity when *q* < 0.05. Duncan’s multiple range test was retained as an exploratory description of group patterns only for outcomes with *q* < 0.05. Results are expressed as mean ± standard error of the mean (SEM), where SEM was calculated from the variation among the six replicate pens within each treatment. Exact unadjusted *p* values and corresponding *q* values are reported together in the tables. Percentage differences from the control are provided for selected outcomes to aid evaluation of magnitude, but practical relevance was interpreted separately from statistical significance.

## 3. Results

### 3.1. Growth Performance and Body Measurements

#### 3.1.1. Growth Performance

AIBW showed no significant differences among all groups (*p* = 0.8872), which indicated the initial body weight of geese was consistent before the trial. BW was markedly higher in all lecithin-supplemented groups relative to CG (*p* = 0.0106). HLG200 and LG100 had greater ADFI than CG, with intermediate values observed in HLG50 and HLG100 (*p* = 0.0335). ADG was uniformly elevated across all lecithin treatments compared with CG (*p* = 0.0081). Regarding FCR, HLG100 and HLG200 exhibited significantly lower values than CG, whereas HLG50 and LG100 showed no statistical difference from CG (*p* = 0.0273) (Table 3).

#### 3.1.2. Body Linear Measurements

None of the body linear measurements remained significant after false-discovery-rate adjustment within this outcome family (all *q* ≥ 0.0659). The nominal differences in body oblique length, keel length, breast depth, shank length, and shank circumference should therefore be regarded as exploratory. (Table 4).

### 3.2. Serum Lipid-Related and Antioxidant Indices

#### 3.2.1. Serum Lipid-Metabolising Enzymes

Serum ALT differed among all experimental groups; HLG200 had the lowest ALT value (*p* = 0.0032). LPL was elevated in all lecithin-supplemented groups relative to CG, with comparable values between HLG200 and LG100 (*p* = 0.0018). SOD and GSH-Px showed no statistical differences across all dietary treatments (*p* = 0.0741 and 0.0683, respectively). MDH was suppressed in all lecithin groups compared with CG, and HLG200 and LG100 had equivalent low MDH values (*p* = 0.0057) (Table 5).

#### 3.2.2. Serum Lipid-Regulating Hormones

Circulating ADPN was substantially higher in all lecithin groups versus CG, and HLG200 had the maximum ADPN content (*p* = 0.0025). LEP was reduced in all supplementation groups, with equivalent minimal values in HLG200 and LG100 (*p* = 0.0011). GLC, T4, and T3 were not statistically altered by diets (*p* = 0.0617, 0.0724, and 0.0816, respectively). INS was increased in all supplemented groups, and HLG200 had higher INS than LG100 (*p* = 0.0009). TRH was suppressed in all hydroxylated lecithin treatments relative to CG; HLG50, HLG100, and HLG200 presented lower TRH concentrations than LG100 (*p* = 0.0043) (Table 6).

#### 3.2.3. Serum Lipoproteins

Serum HDL was elevated in all lecithin-supplemented groups relative to CG, and HLG200 possessed higher HDL than LG100 (*p* = 0.0014). LDL was lower across all treatment groups compared with CG, and HLG200 exhibited the lowest LDL content (*p* = 0.0021). Apo-A1 and Apo-B did not differ statistically among treatments (*p* = 0.0663 and 0.0702, respectively) (Table 7).

#### 3.2.4. Other Serum Lipid-Related Biochemical Indices

GLU and FFA were not statistically modified by dietary lecithin (*p* = 0.0655 and 0.0691, respectively). TC and TG were lower in all lecithin supplementation groups than CG, and LG100 had lower TC and TG values than HLG200 (*p* = 0.0007 and 0.0012, respectively). MDA was suppressed in all supplemented groups relative to CG, with the lowest value detected in HLG200 (*p* = 0.0036). T-GSH was higher in HLG100, HLG200, and LG100 compared with CG, and HLG200 possessed greater T-GSH content than LG100 (*p* = 0.0048) (Table 8).

### 3.3. Carcass Traits and Organ Indices

None of the carcass traits or organ indices remained significant after false-discovery-rate adjustment within this outcome family (all *q* ≥ 0.0869). The nominal differences in breast muscle rate, leg muscle rate, belly fat rate, and spleen index should therefore be interpreted cautiously and require confirmation (Table 9).

### 3.4. Muscle pH, Shear Force, and Muscle-Fibre Morphology

#### 3.4.1. Meat Physical Quality Indicators

Neither breast-muscle nor leg-muscle pH differed among treatments. The nominal treatment effect on breast-muscle shear force did not remain significant after adjustment (*p* = 0.0316, *q* = 0.0506). In contrast, leg-muscle shear force remained different among treatments (*p* = 0.0064, *q* = 0.0128), with lower values in all lecithin-supplemented groups than in CG and the lowest value in LG100 (Table 10).

#### 3.4.2. Muscle-Fibre Morphology

Breast FD and leg FD were smaller in all hydroxylated lecithin treatments compared with CG, and HLG200 had the lowest fibre size for both tissues (*p* = 0.0005 and 0.0008, respectively). Breast FDEN was markedly increased in all supplemented groups relative to CG, with the maximum density observed in HLG200 (*p* = 0.0016). Leg FDEN showed no significant differences across experimental diets (*p* = 0.0771) (Table 11).

### 3.5. Muscle Flavour-Associated Components and Amino Acid Composition

#### 3.5.1. Muscle Flavour Precursor Metabolites

In breast muscle, treatment effects on INO and PL remained significant after adjustment (*p* = 0.0022, *q* = 0.0411; *p* = 0.0295, and *q* = 0.0491, respectively). HLG100 and HLG200 had the highest INO values, whereas HLG100 and LG100 had the highest PL values. The nominal IMF difference did not remain significant after adjustment (*p* = 0.0341, *q* = 0.0512), and TG was unchanged (Table 12).

In leg muscle, the treatment effect on INO remained significant after adjustment (*p* = 0.0307, *q* = 0.0491), with the highest value in HLG100. The nominal IMF difference did not remain significant (*p* = 0.0422, *q* = 0.0596), while TG and PL were unchanged (Table 12).

#### 3.5.2. Muscle Flavour-Associated Amino Acids

In breast muscle, Asp, Ser, Glu, Gly, Ala, and Arg were higher in all lecithin treatments (*p* < 0.05). HLG100 contained the highest umami amino acid levels, with the exception of aspartic acid (Asp). His and Lys showed no inter-group differences (*p* = 0.068 and 0.075, respectively) (Table 13).

In leg muscle, Asp, Ser, Glu, Gly, Ala, and Arg were enriched in supplemented groups (*p* < 0.05). LG100 had the highest Ser and Ala; HLG100 maximised Asp and Gly. His and Lys remained unchanged across treatments (*p* = 0.070 and 0.073, respectively) (Table 13).

### 3.6. Muscle Fatty Acid Composition

#### 3.6.1. Breast Muscle Fatty Acid Profile

After adjustment across the fatty acid outcome family, treatment effects remained for breast muscle C18:2 n6c, C20:4 n6, total PUFA, n-6 PUFA, and the n-6/n-3 ratio (all *q* ≤ 0.0345). The other nominal differences in individual fatty acids did not remain significant (*q* > 0.05). HLG100 generally had the highest values for the FDR-robust outcomes. Total SFA, MUFA, and n-3 PUFA were unchanged (Table 14).

#### 3.6.2. Leg Muscle Fatty Acid Profile

After adjustment across the fatty acid outcome family, treatment effects remained for leg muscle C18:2 n6c, C20:4 n6, and the n-6/n-3 ratio (all *q* ≤ 0.0329). Nominal differences in the other individual fatty acids did not remain significant (*q* > 0.05). Total SFA, MUFA, PUFA, n-3 PUFA, and n-6 PUFA were unchanged (Table 15).

### 3.7. Relative Expression of Lipid Metabolism-Related Genes

**Figure 1:**
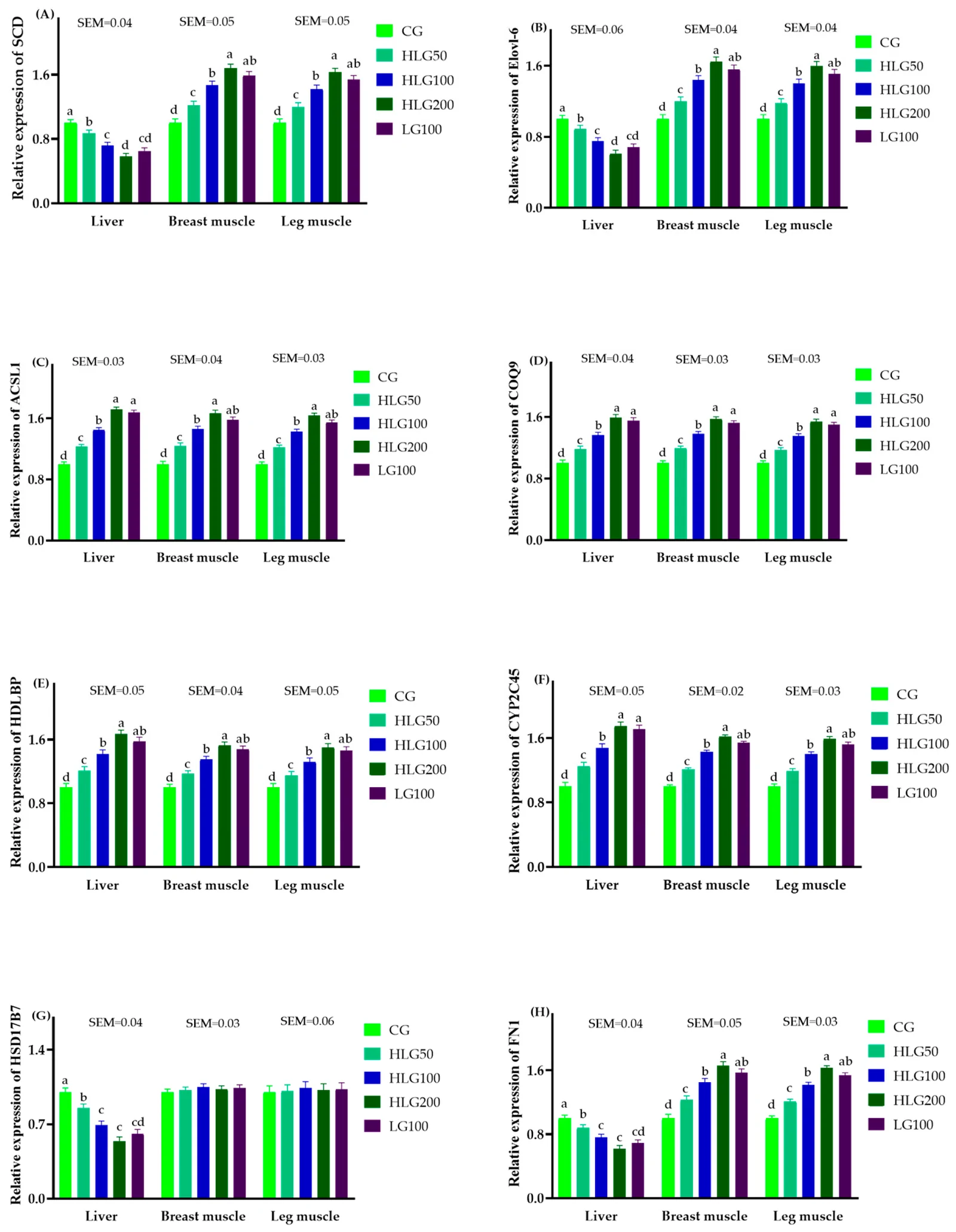
Effects of dietary lecithin treatments on the relative expression of selected lipid metabolism-related genes in the tissues of Jiangnan White geese. (**A**) *SCD*; (**B**) *Elovl6*; (**C**) *ACSL1*; (**D**) *COQ9*; (**E**) *HDLBP*; (**F**) *CYP2C45*; (**G**) *HSD17B7*; and (**H**) *FN1*. Data are presented as means ± SEM (*n* = 6 pens per treatment). Different lowercase letters indicate significant differences among treatments within the same tissue (*p* < 0.05). Relative gene expression was normalised to GAPDH, and the mean expression level of the CG was set to 1.00.

The mRNA abundance of eight lipid metabolism-related genes measured in liver, breast muscle, and leg muscle was markedly altered by dietary lecithin supplementation (Figure 1).

In liver tissue, *SCD*, *Elovl-6*, *HSD17B7* and *FN1* were higher in the control group than in all lecithin treatments, while *ACSL1*, *COQ9*, *HDLBP* and *CYP2C45* were elevated in all lecithin groups (unadjusted *p* = 0.0014–0.0028; *q* = 0.0026–0.0039). HLG200 contained the lowest transcript levels of *SCD*, *Elovl-6*, *HSD17B7* and *FN1*, as well as the highest expression of *ACSL1*, *COQ9*, *HDLBP* and *CYP2C45*.

In breast muscle, *SCD*, *Elovl-6*, *ACSL1*, *COQ9*, *HDLBP*, *CYP2C45* and *FN1* were higher in all lecithin treatments (unadjusted *p* = 0.0017–0.0037; *q* = 0.0031–0.0042). HLG200 contained the highest transcript levels of these seven genes. HSD17B7 showed no inter-group differences (*p* = 0.7826).

In leg muscle, *SCD*, *Elovl-6*, *ACSL1*, *COQ9*, *HDLBP*, *CYP2C45* and *FN1* were enriched in supplemented groups (unadjusted *p* = 0.0019–0.0039; *q* = 0.0033–0.0045). HLG200 maximised the expression of these seven genes. *HSD17B7* remained unchanged across treatments (*p* = 0.8134).

## 4. Discussion

### 4.1. Growth Performance and Potential Nutrient-Utilisation Responses

The present study showed that dietary hydroxylated lecithin was associated with higher growth performance, changes in selected serum lipid-related indices, lower leg-muscle shear force, altered muscle-fibre morphology, changes in several flavour-associated components and fatty acid outcomes, and tissue-specific expression of selected lipid metabolism-related genes. Several nominal findings, including breast-muscle shear force, intramuscular fat, body measurements, and carcass traits, did not remain significant after false-discovery-rate adjustment and should therefore be considered exploratory. The coordinated changes across these endpoints are consistent with differences in lipid utilisation and tissue-specific metabolic responses. However, apparent nutrient digestibility, intestinal lipid absorption, lipid flux, protein abundance, and the activities of key metabolic enzymes were not measured. Therefore, the present findings do not demonstrate that hydroxylated lecithin redirected lipid deposition from abdominal adipose tissue to skeletal muscle, and the proposed changes in lipid partitioning should be regarded as a mechanistic hypothesis requiring functional validation.

The full expression of livestock growth potential hinges on two core determinants: dietary nutrient digestibility and targeted tissue deposition efficiency [30]. As integral structural components of cell membranes, lecithin molecules possess inherent emulsifying activity that breaks dietary fats into stable chylomicrons, expanding the contact surface between lipid droplets and lipases to elevate lipid breakdown efficiency at the point of digestion [31,32]. Numerous feeding trials with broilers have confirmed that exogenous phospholipid additives can boost lipid digestibility and dietary metabolizable energy [33,34,35]. Zhang et al. [33] observed that lysophosphatidylcholine supplementation raised the total tract apparent digestibility of C16:0 and other fatty acids and improved body weight gain in starter broilers. In line with these findings, Boontiam et al. [34] further reported that graded lysophospholipid supplementation ranging from 0.05% to 0.15% linearly enhanced broiler growth performance as well as crude fat and protein digestibility throughout the entire rearing cycle. Moreover, Jansen et al. [35] supplemented 250 ppm soybean or rapeseed lysolecithin and verified that such additives increased dry matter digestibility, nitrogen retention and AMEn (apparent metabolizable energy, nitrogen-corrected) in 24–28-day-old broilers receiving lard-containing diets. Yet soybean lecithin suffers intrinsic limitations in water solubility and dispersibility; its emulsifying performance fluctuates sharply with dietary fat concentration and animal developmental stage, yielding inconsistent growth-promoting effects in adult poultry fed high-fat diets [36]. Covalent attachment of hydroxyl groups to phospholipid carbon chains elevates molecular hydrophilicity and interfacial activity, enabling more complete intestinal fat emulsification and concurrent increases in epithelial transmembrane absorption of phospholipids, cholesterol and all liposoluble vitamins. Improved molecular polarity drives enhanced interfacial activity and robust chylomicron formation, with these molecular-scale alterations translating into a marked overall rise in lipid utilisation efficiency [37,38]. At the same inclusion level of 100 mg/kg, hydroxylated and conventional soybean lecithin produced overlapping responses for several growth and serum endpoints, whereas HLG100 produced higher values for selected muscle flavour-associated components, including inosine monophosphate. These findings indicate partly distinct response profiles between the two lecithin sources rather than a uniform advantage of hydroxylated lecithin across all measured outcomes. The partly distinct response profiles may reflect differences in the physicochemical properties of the two lecithin sources. However, the equal-dose comparison did not demonstrate a consistent advantage of hydroxylated lecithin over conventional soybean lecithin across growth-, serum-, and meat-related outcomes. Direct measurements of emulsification, digestibility, and lipid absorption are required before these differences can be attributed to the greater hydrophilicity of hydroxylated lecithin.

### 4.2. Serum Lipid-Related and Antioxidant Responses

Stepwise remodelling of systemic lipid homeostasis follows gains in dietary nutrient utilisation, with serum lipid profiles and energy-regulating endocrine hormones serving as the most accessible biomarkers for dissecting this regulatory network [39]. Hydroxylated lecithin was associated with marked changes in circulating lipid fractions and lipid-related hormones, suggesting that its effects may extend beyond intestinal lipid digestion. Elevated HDL, the primary vehicle for reverse cholesterol transport, facilitates efficient clearance of peripheral excess cholesterol to the liver for catabolism, while concurrent reductions in LDL mitigate risks of systemic lipid overload [40]. The lower serum triglyceride and total cholesterol concentrations are consistent with altered systemic lipid handling. However, because lipid turnover and metabolic flux were not measured, these changes cannot be interpreted as direct evidence of accelerated lipid catabolism or reduced whole-body fat accumulation [41]. Classic models describing phospholipid-mediated lipoprotein assembly and very-low-density lipoprotein secretion may partly explain the coordinated shifts in HDL and LDL observed in the present study [42]. Cellular and animal models consistently report lecithin-mediated upregulation of adiponectin, improved peripheral insulin sensitivity and accelerated fatty acid β-oxidation [43]. The marked adiponectin upregulation observed in this study is consistent with these previous observations and further supports the potential involvement of lipid metabolic regulation. Previous studies mainly described changes in circulating lipid and hormone concentrations [44,45]. In the present study, changes in circulating biomarkers were accompanied by tissue-specific differences in candidate-gene expression. These parallel responses generate a hypothesis that hydroxylated lecithin may influence tissue-specific lipid metabolism, but they do not demonstrate altered lipid partitioning among tissues. However, the causal relationships between these physiological responses and the observed transcriptional changes remain to be established through functional validation.

The coordinated alterations in adiponectin, leptin, and insulin suggest potential interactions among endocrine regulators involved in systemic energy homeostasis. Adiponectin, a master adipokine governing fatty acid oxidation, typically rises alongside enhanced peripheral insulin sensitivity, directing greater quantities of free fatty acids toward mitochondrial oxidative catabolism [46]. Fluctuations in leptin directly reflect total body fat reserves and systemic energy balance, while insulin acts as the central hub coordinating carbohydrate and lipid metabolism, mediating targeted nutrient deposition within growing tissues [47,48]. The coordinated hormonal changes observed in the present study were accompanied by the absence of excessive abdominal fat deposition, suggesting that hydroxylated lecithin did not promote undesirable fat accumulation. These endocrine responses may be associated with differences in systemic lipid utilisation. However, because the nominal differences in intramuscular fat did not remain significant after false-discovery-rate adjustment, the present data do not establish enhanced intramuscular lipid deposition or altered nutrient partitioning. The lower MDA concentration and higher total glutathione level are consistent with an improved circulating redox profile. Nevertheless, these serum biomarkers do not establish that hydroxylated lecithin directly prevented cellular oxidative injury or modified mitochondrial antioxidant pathways. Because membrane phospholipids are susceptible to oxidative damage, the improved antioxidant status observed in the present study may have contributed to maintaining membrane integrity and cellular metabolic function. Nevertheless, whether hydroxylated lecithin directly modulates mitochondrial function or coenzyme Q-dependent antioxidant pathways remains to be determined.

### 4.3. Meat Quality, Muscle Microstructure, and Flavour-Associated Components

From an industrial translation perspective, improved meat quality represents the most practically valuable endpoint of nutritional manipulation. Intramuscular fat dictates poultry tenderness, juiciness and flavour release potential, yet excessive abdominal fat deposition erodes carcass commercial value [49]. Strategies to selectively enrich intramuscular fat while suppressing ectopic fat storage have therefore remained a central research priority within meat poultry nutrition [50,51]. Geese fed hydroxylated lecithin exhibited higher intramuscular fat content and changes in tenderness-related muscle characteristics. Leg-muscle shear force was lower at all tested supplementation levels, whereas breast-muscle shear force was significantly lower only in the 200 mg/kg group. Hydroxylated lecithin supplementation was also associated with smaller fibre diameter in breast and leg muscles and greater fibre density in breast muscle, while abdominal fat rate did not differ significantly from that of the control group. Neither the nominal increase in intramuscular fat nor the differences in abdominal fat rate remained significant after multiplicity adjustment. The present findings therefore do not support preferential lipid deposition in muscle over abdominal adipose tissue. Although intramuscular fat can reduce mechanical resistance between muscle-fibre bundles [48], this mechanism cannot be used to explain the lower leg-muscle shear force observed here without confirmatory measurements of lipid deposition and muscle structure. Smaller fibre diameter and greater fibre density have also been associated with a finer muscle structure and greater tenderness [52,53]. Consistent with these associations, Huo et al. [54] reported that differences in muscle-fibre diameter, density, and cross-sectional area were correlated with shear force, pH, intramuscular fat, and other meat-quality characteristics in fast- and slow-growing ducks. Weng et al. [55] similarly showed that age-related increases in muscle-fibre diameter and cross-sectional area were accompanied by changes in the physical and compositional characteristics of Yangzhou goose meat. Collectively, these studies support an association between muscle-fibre morphology and tenderness-related traits. However, the present study did not include sensory evaluation or direct measurements of lipid flux; therefore, the contribution of the observed structural changes to eating quality and tissue lipid distribution requires further investigation.

Beyond muscle microstructure, sensory and nutritional meat quality gains rely heavily on sustained endogenous accumulation of flavour precursors [25]. Hydroxylated lecithin was associated with changes in muscle inosine monophosphate, several flavour-associated amino acids, and selected fatty acid outcomes. After multiplicity adjustment, the most robust fatty acid responses involved linoleic acid, arachidonic acid, total PUFA and n-6 PUFA in breast muscle, together with the n-6/n-3 ratio. Inosine monophosphate constitutes the primary flavour-active nucleotide in poultry meat, generating robust umami synergism when paired with glutamate, a chemical interaction thoroughly validated in avian flavour chemistry research [26]. Glutamate, aspartate, glycine and alanine deliver intrinsic sweet and savoury base notes, while participating in Maillard reactions during thermal processing to yield abundant volatile aromatic compounds [27,28]. Polyunsaturated fatty acids serve as precursor substances for aldehydes, ketones and alcohols that generate distinctive flavour profiles [56]. Existing research has comprehensively clarified the molecular pathways connecting the oxidative breakdown of polyunsaturated fatty acids to unique aroma characteristics of different poultry varieties [56,57]. The aggregated data further indicate that hydroxylated lecithin, particularly at 100 mg/kg, increased the concentrations of several individual PUFAs, including linoleic acid, arachidonic acid, and docosahexaenoic acid, and elevated total PUFA in breast muscle without significant alterations in total SFA or n-3 PUFA. Notably, the rise in total PUFA was predominantly driven by n-6 polyunsaturated fractions, which led to a notable increase in the n-6/n-3 ratio. Such compositional changes improve the abundance of flavour precursors, yet the elevated PUFA level alone cannot be simply judged as an overall upgrade of meat nutritional quality, and the shifted n-6/n-3 ratio requires prudent nutritional evaluation rather than being recognised as a favourable nutritional trait. Although the n-6/n-3 ratio increased following hydroxylated lecithin supplementation, this change should be interpreted cautiously because the nutritional value of meat depends on the overall balance of individual fatty acids rather than the n-6/n-3 ratio alone. While docosahexaenoic acid (DHA) content was slightly elevated, the overall PUFA increment was dominated by n-6 fatty acids. Thus, hydroxylated lecithin merely reshaped muscle fatty acid composition, and the compositional shift cannot be described as an unequivocal improvement of meat nutritional quality. Nominal differences were also observed for DHA and several other individual fatty acids, but these findings did not remain significant after false-discovery-rate adjustment and require independent confirmation. The concurrent enrichment of flavour precursors and partial long-chain polyunsaturated fatty acids suggests that hydroxylated lecithin may provide a more favourable biochemical basis for meat flavour development, yet the modification of fatty acid composition (mainly increased n-6 PUFA and higher n-6/n-3 ratio) cannot be regarded as an absolute optimisation of meat nutritional properties. Among the tested doses, 100 mg/kg produced the highest numerical values for several flavour-associated endpoints, including inosine monophosphate and selected amino acids. However, the intramuscular-fat response did not remain significant after multiplicity adjustment, and the present design does not establish an optimal biological or commercial dose. The endpoint-specific response patterns should therefore be confirmed in dose–response studies with prespecified primary outcomes. These findings suggest that different supplementation levels of hydroxylated lecithin may produce distinct physiological responses, providing useful information for optimising dietary inclusion levels under commercial production conditions.

### 4.4. Tissue-Specific Expression of Lipid Metabolism-Related Genes

The coordinated physiological changes observed in the present study were accompanied by tissue-specific differences in the expression of lipid metabolism-related genes in the liver and skeletal muscle [58,59,60]. Previous studies have primarily focused on the regulation of lipid metabolism within individual tissues. In contrast, the present study identified distinct patterns of lipid metabolism-related gene expression in the liver and skeletal muscle, suggesting tissue-specific differences in lipid metabolism-related gene expression following hydroxylated lecithin supplementation. The selected genes represent complementary biological processes involved in lipid metabolism, including ACSL1, SCD, Elovl6, HSD17B7, HDLBP, COQ9, FN1, and CYP2C45. Together, these genes provide an integrated overview of tissue-specific transcriptional responses associated with hydroxylated lecithin supplementation rather than focusing on a single metabolic process. In the liver, the expression of *SCD*, *Elovl6*, *HSD17B7*, and *FN1* was downregulated, whereas *ACSL1*, *COQ9*, *HDLBP*, and *CYP2C45* were upregulated. In breast and leg muscle, seven of the eight candidate genes showed expression patterns that differed from their hepatic responses, whereas *HSD17B7* was unchanged in both muscle tissues. These contrasting expression patterns are consistent with tissue-specific transcriptional responses related to lipid metabolism. However, because tissue lipid flux and protein abundance were not measured and the nominal IMF differences did not survive multiplicity adjustment, the expression data cannot establish differences in lipid deposition among tissues. Because *SCD* and *Elovl6* are involved in fatty acid synthesis, their reduced expression in the liver may indicate a lower capacity for hepatic lipogenesis. Previous studies have shown that *ACSL1*, *HDLBP*, and *CYP2C45* participate in fatty acid activation, lipoprotein metabolism, and lipid oxidation, respectively. Their increased expression observed in the present study may therefore be associated with altered hepatic lipid metabolism. Collectively, these expression changes suggest altered hepatic lipid metabolic activity. The increased expression of lipid metabolism-related genes in skeletal muscle may have facilitated greater lipid utilisation and intramuscular lipid deposition, although the underlying metabolic fluxes were not directly examined in the present study. Because *COQ9* participates in mitochondrial coenzyme Q biology, its altered expression provides a hypothesis for future investigation. The present mRNA and serum biomarker data are insufficient to establish a functional link between *COQ9* expression and antioxidant status. However, this possibility requires further experimental verification. Collectively, these findings suggest that hydroxylated lecithin was associated with coordinated changes in tissue-specific gene expression, which may contribute to differences in lipid metabolism among tissues. These observations provide a basis for future studies investigating the role of modified phospholipids in precision livestock nutrition.

### 4.5. Study Limitations and Future Directions

Despite the breadth of phenotypic and molecular measurements included in this study, its mechanistic interpretation remains constrained by the experimental design. The large endpoint panel created a substantial multiplicity burden. False-discovery-rate control reduced this risk, but several nominal findings, including all body-measurement and carcass outcomes, breast-muscle shear force, intramuscular fat, and many individual fatty acids, did not remain significant and should be treated as exploratory. Statistical significance was also not assumed to establish commercial importance. For example, the nominal increase in breast-muscle IMF from 3.15% to 3.35% at 100 mg/kg represented an absolute change of 0.20 percentage points, or 6.3% relative to CG, and did not survive adjustment. By contrast, ADG increased by 12.5%, and FCR decreased by 9.3% at 100 mg/kg, whereas leg-muscle shear force decreased by 28.0%; these larger changes may be more relevant biologically, but their economic and sensory importance requires independent validation under commercial conditions. The increase in breast-muscle total PUFA was substantial, but it was driven mainly by n-6 PUFA and accompanied by a higher n-6/n-3 ratio, preventing a simple claim of improved nutritional value.

The molecular evidence was limited to the mRNA abundance of a hypothesis-driven panel of eight genes, without corresponding measurements of protein abundance, key enzyme activities, or intracellular lipid transport. The panel sampled complementary lipid–metabolism and tissue-remodelling functions but was not intended to provide pathway-wide coverage. Moreover, qPCR normalisation relied on GAPDH alone, and its stability across tissues and treatments was not formally validated against additional reference genes. These constraints reduce confidence in the molecular component, so the transcriptional results should be considered supportive and hypothesis-generating. Apparent digestibility of crude fat, protein, and individual fatty acids was not determined, and intestinal lipase activity, micelle formation, and lipid-transporter abundance were not assessed. Consequently, the growth and feed-efficiency responses cannot be directly attributed to enhanced nutrient digestion or absorption. Potential intestinal contributions also remain unclear because mucosal-barrier function, bile acid metabolism, and gut microbial composition were not examined. The use of female Jiangnan White geese during a single 64-day production cycle further limits extrapolation to males, other breeds, and different growth stages. Future studies should predefine a limited set of primary outcomes, report confidence intervals and validated effect-size measures from raw pen-level data, validate multiple reference genes, and combine digestibility and intestinal lipid-transport measurements with proteomics, lipidomics, gut metagenomics, and metabolic-flux analyses. Replication across sexes, breeds, growth stages, and production settings is needed to determine practical benefit.

## 5. Conclusions

After control of the false discovery rate, dietary hydroxylated lecithin remained associated with higher growth performance, selected serum lipid-related and antioxidant indices, lower leg-muscle shear force, altered muscle-fibre morphology, changes in inosine mono-phosphate and several flavour-associated amino acids, selected fatty acid outcomes, and tissue-specific expression of the candidate genes in female Jiangnan White geese. At 100 mg/kg, ADG increased from 49.32 to 55.50 g/day and FCR decreased from 3.87 to 3.51, corresponding to relative changes of 12.5% and 9.3%, respectively. Nominal differences in body measurements, carcass traits, breast-muscle shear force, intramuscular fat, and many individual fatty acids did not withstand adjustment and should not be considered confirmed effects. The increase in breast-muscle PUFA was driven mainly by n-6 PUFA and accompanied by a higher n-6/n-3 ratio, so improved nutritional value cannot be inferred. The candidate-gene results support tissue-specific transcriptional responses but do not demonstrate lipid redistribution or causality. Independent studies using prespecified primary outcomes, validated reference genes, digestibility measurements, protein and enzyme assays, and metabolic-flux analysis are required to establish practical relevance and mechanism.

## Figures and Tables

**Table 1:** Composition and nutrient levels (air-dry basis, g/100 g diet) of basal diets for Jiangnan White geese at three growth phases.

Ingredients	1–21 d	22–42 d	43–64 d
Corn	40.00	42.00	44.00
Corn gluten meal	8.00	7.00	6.00
Soybean oil	1.50	1.20	0.90
Soybean meal	19.00	17.00	15.00
Wheat bran	10.00	10.00	10.00
Defatted rice bran	16.25	17.25	18.25
Dicalcium phosphate	0.90	0.90	0.90
Limestone	3.60	3.90	4.20
Sodium chloride	0.35	0.35	0.35
Premix ^1^	0.40	0.40	0.40
Total, g/100 g	100.00	100.00	100.00
Nutrient levels			
Metabolizable energy (ME) ^2^, MJ/kg	12.04	11.04	10.04
Crude protein (CP) ^3^, %	20.00	18.00	16.00
Crude fibre (CF) ^3^, %	5.00	8.00	11.00
Calcium (Ca) ^3^, %	0.90	0.90	0.90
Total phosphorus (TP) ^3^, %	0.60	0.50	0.45
Available phosphorus (AP) ^3^, %	0.38	0.32	0.29
Lysine (Lys) ^3^, %	1.10	0.90	0.80
Methionine (Met) ^3^, %	0.60	0.50	0.50

^1^ The vitamin–mineral premix provided the following nutrients per kilogram of premix: vitamin A 15,000.00 IU, vitamin D_3_ 5300.00 IU, vitamin E 100.00 mg, vitamin K 4.00 mg, thiamine 2.00 mg, vitamin B_2_ (riboflavin) 1200.00 mg, pantothenic acid 50.00 mg, nicotinic acid 10.25 mg, pyridoxine 3.85 mg, vitamin B_12_ 0.10 mg, folic acid 2.00 mg, biotin 0.21 mg, and vitamin C 200.00 mg; Mn (as manganese sulfate) 80.00 mg, Fe (as ferrous sulfate) 60.00 mg, Cu (as copper sulfate) 20.00 mg, I (as potassium iodide) 3.00 mg, Se (as sodium selenite) 0.50 mg, Zn (as zinc sulfate) 100.00 mg, and choline (as choline chloride) 1400.00 mg. ^2^ Calculated values. ^3^ Analysed values determined by chemical analysis.

**Table 2:** Primer sequences of lipid metabolism-related genes.

Gene	Primer Sequence (5′ → 3′)	Ampliconsize (bp)	Accession No.
*SCD*	F: CACCTGGCTAGTGAACAG R: AACTCACTGGTGGAGTAGTC	159	HO224431
*Elovl-6*	F: TGGACCTGTCAGCAAATTC R: AGCCACCATGTCCTTGTAG	169	HO224432
*HDLBP*	F: TGAGTTCAAGGTGGATATTCG R: TGTCCACCACATCTGCAAG	146	HO224433
*COQ9*	F: CTGAACATGGCTGGACG R: GTTGCACTGAGACACAAAGTG	128	HO224438
*ACSL1*	F: TAGCCCAGGGAGAGTACATAG R: GGTTTCGGGATCAGGTACC	137	HO224439
*HSD17B7*	F: GTCACGACTCATCTGGAC R: ACAACACTGGAATACAGACC	174	HO224440
*FN1*	F: GGCAACTGGACCTGTTC R: CTCCAGCGCAGAATGTAC	117	HO224441
*CYP2C45*	F: CAGAATGGCACCTTTAGG R: GGAGTAAGAATATCTCCATGC	103	HO224442
*GAPDH*	F: CAGATGCAGGTGCTGAGTAT R: GACACCCATCACAAACATG	143	HO224443

SCD, stearoyl-coenzyme A desaturase; Elovl-6, ELOVL family member 6; ACSL1, long-chain acyl-coenzyme A synthetase 1; COQ9, coenzyme Q9; HDLBP, high-density lipoprotein-binding protein; CYP2C45, cytochrome P450 2C45; HSD17B7, hydroxysteroid (17-β) dehydrogenase 7; FN1, fibronectin 1.

**Table 3:** Effects of dietary lecithin treatments on growth performance of Jiangnan White geese.

Items	CG	HLG50	HLG100	HLG200	LG100	SEM	*p*-Value	*q*-Value
AIBW, g	119	118	120	120	119	1.32	0.8872	0.8872
BW, g	3291 ^b^	3570 ^a^	3674 ^a^	3662 ^a^	3582 ^a^	22.16	0.0106	0.0265
ADFI, g/d	192 ^b^	196 ^ab^	195 ^ab^	197 ^a^	197 ^a^	0.94	0.0335	0.0419
ADG, g/d	49.32 ^b^	53.91 ^a^	55.50 ^a^	55.46 ^a^	54.21 ^a^	0.61	0.0081	0.0265
FCR, kg/kg	3.87 ^a^	3.63 ^ab^	3.51 ^b^	3.56 ^b^	3.64 ^ab^	0.07	0.0273	0.0419

AIBW, average initial body weight; BW, body weight; ADFI, average daily feed intake; ADG, average daily gain; FCR, feed conversion ratio. ^a,b^ *p* and *q* are the unadjusted omnibus ANOVA and Benjamini–Hochberg-adjusted values, respectively. Within rows with *q* < 0.05, different lowercase superscripts describe Duncan post hoc group patterns (*p* < 0.05). CG (*n* = 6), control group; HLG50 (*n* = 6), basal diet with 50 mg/kg hydroxylated lecithin; HLG100 (*n* = 6), basal diet with 100 mg/kg hydroxylated lecithin; HLG200 (*n* = 6), basal diet with 200 mg/kg hydroxylated lecithin; LG100 (*n* = 6), basal diet with 100 mg/kg soybean lecithin; SEM, standard error of the mean.

**Table 4:** Effects of hydroxylated lecithin on body sizes of Jiangnan White geese.

Items	CG	HLG50	HLG100	HLG200	LG100	SEM	*p*-Value	*q*-Value
Semi-submerged length, cm	66.83	65.50	66.58	66.50	65.08	0.56	0.7914	0.7914
Body oblique length, cm	27.42	27.00	27.33	26.17	27.83	0.21	0.0412	0.0659
Keel length, cm	14.42	13.58	14.92	13.83	13.67	0.18	0.0267	0.0659
Breast depth, cm	9.00	9.37	9.23	9.49	9.44	0.07	0.0389	0.0659
Breast width, cm	11.16	11.45	11.95	11.76	11.39	0.13	0.4356	0.5808
Shank length, cm	7.71	7.53	8.35	7.99	8.13	0.08	0.0147	0.0659
Shank circumference, cm	4.75	4.83	5.00	5.00	4.95	0.04	0.0219	0.0659
Pelvic width, cm	7.70	7.31	7.46	7.12	7.13	0.11	0.6720	0.7680

*p* and *q* represent the unadjusted omnibus ANOVA *p* value and the Benjamini–Hochberg-adjusted value, respectively. No outcome remained significant after false discovery rate adjustment at *q* < 0.05. CG (*n* = 6), control group; HLG50 (*n* = 6), basal diet with 50 mg/kg hydroxylated lecithin; HLG100 (*n* = 6), basal diet with 100 mg/kg hydroxylated lecithin; HLG200 (*n* = 6), basal diet with 200 mg/kg hydroxylated lecithin; LG100 (*n* = 6), basal diet with 100 mg/kg soybean lecithin; SEM, standard error of the mean.

**Table 5:** Effects of dietary lecithin treatments on enzyme activity related to lipid metabolism in serum of Jiangnan White geese.

Items	CG	HLG50	HLG100	HLG200	LG100	SEM	*p*-Value	*q*-Value
ALT, U/L	12.52 ^a^	10.32 ^b^	8.17 ^c^	6.49 ^d^	8.10 ^c^	0.46	0.0032	0.0078
LPL, μmolFFA/mL·h	1.97 ^d^	2.03 ^c^	2.24 ^b^	2.37 ^a^	2.35 ^a^	0.04	0.0018	0.0066
SOD, U/mL	77.39	79.42	88.73	89.67	89.80	2.15	0.0741	0.0776
GSH-Px, U/mL	829	855	875	892	880	4.76	0.0683	0.0776
MDH, U/mL	48.76 ^a^	42.69 ^b^	42.91 ^b^	36.96 ^c^	36.19 ^c^	1.34	0.0057	0.0096

ALT, alanine aminotransferase; LPL, lipoprotein lipase; SOD, superoxide dismutase; GSH-Px, glutathione peroxidase; MDH, malate dehydrogenase. ^a,b,c,d^ *p* and *q* are the unadjusted omnibus ANOVA and Benjamini–Hochberg-adjusted values, respectively. Within rows with *q* < 0.05, different lowercase superscripts describe Duncan post hoc group patterns (*p* < 0.05). CG (*n* = 6), control group; HLG50 (*n* = 6), basal diet with 50 mg/kg hydroxylated lecithin; HLG100 (*n* = 6), basal diet with 100 mg/kg hydroxylated lecithin; HLG200 (*n* = 6), basal diet with 200 mg/kg hydroxylated lecithin; LG100 (*n* = 6), basal diet with 100 mg/kg soybean lecithin; SEM, standard error of the mean.

**Table 6:** Effects of dietary lecithin treatments on hormone levels related to lipid metabolism in serum of Jiangnan White geese.

Items	CG	HLG50	HLG100	HLG200	LG100	SEM	*p*-Value	*q*-Value
ADPN, mg/L	6.00 ^d^	8.07 ^c^	10.29 ^b^	14.32 ^a^	11.82 ^ab^	0.56	0.0025	0.0069
LEP, ng/mL	7.56 ^a^	5.31 ^b^	3.90 ^c^	3.10 ^d^	3.12 ^d^	0.41	0.0011	0.0062
GLC, pg/mL	98.85	88.14	80.45	78.22	75.26	2.64	0.0617	0.0776
INS, μIU/mL	6.00 ^d^	8.56 ^c^	11.90 ^b^	14.69 ^a^	12.03 ^b^	0.68	0.0009	0.0062
T4, ng/mL	22.81	19.18	16.31	16.40	14.01	0.74	0.0724	0.0776
T3, ng/mL	1.17	1.15	1.14	1.23	1.14	0.01	0.0816	0.0816
TRH, pg/mL	20.44 ^a^	9.11 ^e^	12.06 ^d^	14.36 ^c^	16.84 ^b^	0.95	0.0043	0.0086

ADPN, adiponectin; LEP, leptin; GLC, glucagon; INS, insulin; T4, thyroxine; T3, triiodothyronine; TRH, thyrotropin releasing hormone. ^a,b,c,d,e^ *p* and *q* are the unadjusted omnibus ANOVA and Benjamini–Hochberg-adjusted values, respectively. Within rows with *q* < 0.05, different lowercase superscripts describe Duncan post hoc group patterns (*p* < 0.05). CG (*n* = 6), control group; HLG50 (*n* = 6), basal diet with 50 mg/kg hydroxylated lecithin; HLG100 (*n* = 6), basal diet with 100 mg/kg hydroxylated lecithin; HLG200 (*n* = 6), basal diet with 200 mg/kg hydroxylated lecithin; LG100 (*n* = 6), basal diet with 100 mg/kg soybean lecithin; SEM, standard error of the mean.

**Table 7:** Effects of dietary lecithin treatments on lipoprotein levels related to lipid metabolism in serum of Jiangnan White geese.

Items	CG	HLG50	HLG100	HLG200	LG100	SEM	*p*-Value	*q*-Value
HDL, mmol/L	1.66 ^d^	1.75 ^c^	1.97 ^b^	2.17 ^a^	2.04 ^b^	0.02	0.0014	0.0062
LDL, mmol/L	1.74 ^a^	1.58 ^b^	1.35 ^c^	1.30 ^d^	1.34 ^c^	0.04	0.0021	0.0066
Apo-A1, g/L	0.15	0.15	0.18	0.21	0.24	0.03	0.0663	0.0776
Apo-B, g/L	0.72	0.69	0.66	0.60	0.63	0.02	0.0702	0.0773

HDL, high-density lipoprotein; LDL, low-density lipoprotein; Apo-A1, apolipoprotein A1; Apo-B, apolipoprotein B. ^a,b,c,d^ *p* and *q* are the unadjusted omnibus ANOVA and Benjamini–Hochberg-adjusted values, respectively. Within rows with *q* < 0.05, different lowercase superscripts describe Duncan post hoc group patterns (*p* < 0.05). CG (*n* = 6), control group; HLG50 (*n* = 6), basal diet with 50 mg/kg hydroxylated lecithin; HLG100 (*n* = 6), basal diet with 100 mg/kg hydroxylated lecithin; HLG200 (*n* = 6), basal diet with 200 mg/kg hydroxylated lecithin; LG100 (*n* = 6), basal diet with 100 mg/kg soybean lecithin; SEM, standard error of the mean.

**Table 8:** Effects of dietary lecithin treatments on other biochemical indicator levels related to lipid metabolism in serum of Jiangnan White geese.

Items	CG	HLG50	HLG100	HLG200	LG100	SEM	*p*-Value	*q*-Value
GLU, mmol/L	14.80	11.50	11.70	9.56	11.49	0.82	0.0655	0.0773
TC, mmol/L	4.98 ^a^	4.52 ^b^	3.82 ^c^	3.45 ^d^	3.20 ^e^	0.11	0.0007	0.0062
TG, mmol/L	1.91 ^a^	1.75 ^b^	1.45 ^c^	1.38 ^d^	1.29 ^e^	0.04	0.0012	0.0032
MDA, nmol/mL	4.96 ^a^	4.32 ^b^	4.28 ^b^	3.86 ^d^	4.00 ^c^	0.14	0.0036	0.0079
FFA, mmol/L	0.41	0.44	0.47	0.50	0.50	0.01	0.0691	0.0776
T-GSH, μmol/L	10.62 ^c^	10.79 ^c^	13.43 ^b^	15.70 ^a^	13.58 ^b^	0.51	0.0048	0.0088

GLU, glucose; TC, total cholesterol; TG, triglycerides; MDA, malondialdehyde; FFA, free fatty acid; T-GSH, total glutathione. ^a,b,c,d,e^ *p* and *q* are the unadjusted omnibus ANOVA and Benjamini–Hochberg-adjusted values, respectively. Within rows with *q* < 0.05, different lowercase superscripts describe Duncan post hoc group patterns *(p* < 0.05). CG (*n* = 6), control group; HLG50 (*n* = 6), basal diet with 50 mg/kg hydroxylated lecithin; HLG100 (*n* = 6), basal diet with 100 mg/kg hydroxylated lecithin; HLG200 (*n* = 6), basal diet with 200 mg/kg hydroxylated lecithin; LG100 (*n* = 6), basal diet with 100 mg/kg soybean lecithin; SEM, standard error of the mean.

**Table 9:** Effects of hydroxylated lecithin on slaughter performance and visceral organ indices of Jiangnan White geese.

Items	CG	HLG50	HLG100	HLG200	LG100	SEM	*p*-Value	*q*-Value
SR, %	86.57	87.60	85.97	87.67	88.22	2.35	0.9217	0.9543
Semi-ER, %	79.98	79.93	78.80	79.16	79.74	2.41	0.9543	0.9566
ER, %	70.04	70.62	68.68	69.69	69.41	1.86	0.9072	0.9543
BR, %	7.50	8.84	8.99	7.73	8.08	0.18	0.0163	0.0896
LR, %	12.43	12.20	17.57	17.53	14.82	0.57	0.0079	0.0869
BFR, %	3.34	3.99	4.53	4.20	5.34	0.26	0.0374	0.1114
CI, %	0.66	0.65	0.62	0.58	0.66	0.06	0.8466	0.9522
LI, %	2.12	2.17	2.25	2.34	2.34	0.08	0.7531	0.9536
SI, %	0.08	0.11	0.11	0.13	0.11	0.01	0.0405	0.1121
GI, %	3.66	3.56	3.23	3.35	3.00	0.16	0.5824	0.9544
GSI, %	0.38	0.35	0.35	0.34	0.33	0.01	0.7139	0.9537

SR, slaughter rate; semi-ER, semi-eviscerated rate; ER, eviscerated rate; BR, breast muscle rate; LR, leg muscle rate; BFR, belly fat rate; CI, cardiac index; LI, liver index; SI, spleen index; GI, gizzard index; GSI, glandular stomach index. *p* and *q* are the unadjusted omnibus ANOVA and Benjamini–Hochberg-adjusted values, respectively. Within rows with *q* < 0.05, different lowercase superscripts describe Duncan post hoc group patterns (*p* < 0.05). CG (*n* = 6), control group; HLG50 (*n* = 6), basal diet with 50 mg/kg hydroxylated lecithin; HLG100 (*n* = 6), basal diet with 100 mg/kg hydroxylated lecithin; HLG200 (*n* = 6), basal diet with 200 mg/kg hydroxylated lecithin; LG100 (*n* = 6), basal diet with 100 mg/kg soybean lecithin; SEM, standard error of the mean.

**Table 10:** Effects of dietary lecithin treatments on muscle pH and shear force in Jiangnan White geese.

Items	CG	HLG50	HLG100	HLG200	LG100	SEM	*p*-Value	*q*-Value
Breast muscle pH	5.89	5.81	5.93	5.90	5.90	0.21	0.9136	0.9472
Leg muscle pH	5.90	5.89	5.90	5.88	5.91	0.06	0.9472	0.9472
BMSF, N	60.58	54.13	51.88	43.85	52.66	3.04	0.0316	0.0506
LMSF, N	49.98 ^a^	41.83 ^b^	36.00 ^c^	38.57 ^b^	32.68 ^d^	1.36	0.0064	0.0128

BMSF, breast muscle shear force; LMSF, leg-muscle shear force. ^a,b,c,d^ *p* and *q* are the unadjusted omnibus ANOVA and Benjamini–Hochberg-adjusted values, respectively. Within rows with *q* < 0.05, different lowercase superscripts describe Duncan post hoc group patterns (*p* < 0.05). CG (*n* = 6), control group; HLG50 (*n* = 6), basal diet with 50 mg/kg hydroxylated lecithin; HLG100 (*n* = 6), basal diet with 100 mg/kg hydroxylated lecithin; HLG200 (*n* = 6), basal diet with 200 mg/kg hydroxylated lecithin; LG100 (*n* = 6), basal diet with 100 mg/kg soybean lecithin; SEM, standard error of the mean.

**Table 11:** Effects of dietary lecithin treatments on muscle fibre diameter and density of Jiangnan White geese.

Items	CG	HLG50	HLG100	HLG200	LG100	SEM	*p*-Value	*q*-Value
Breast muscle FD, μm	70.73 ^a^	60.65 ^b^	55.92 ^c^	40.63 ^e^	50.73 ^d^	2.34	0.0005	0.0032
Leg muscle FD, μm	110 ^a^	101 ^b^	92.83 ^c^	73.34 ^e^	83.80 ^d^	2.16	0.0008	0.0026
Breast muscle FDEN, N/mm^2^	107 ^d^	189 ^c^	193 ^c^	334 ^a^	303 ^b^	9.85	0.0016	0.0043
Leg muscle FDEN, N/mm^2^	72.84	84.44	95.31	95.21	106	3.27	0.0771	0.1028

FD, fibre diameter; FDEN, fibre density. ^a,b,c,d,e^ *p* and *q* are the unadjusted omnibus ANOVA and Benjamini–Hochberg-adjusted values, respectively. Within rows with *q* < 0.05, different lowercase superscripts describe Duncan post hoc group patterns (*p* < 0.05). CG (*n* = 6), control group; HLG50 (*n* = 6), basal diet with 50 mg/kg hydroxylated lecithin; HLG100 (*n* = 6), basal diet with 100 mg/kg hydroxylated lecithin; HLG200 (*n* = 6), basal diet with 200 mg/kg hydroxylated lecithin; LG100 (*n* = 6), basal diet with 100 mg/kg soybean lecithin; SEM, standard error of the mean.

**Table 12:** Effects of dietary lecithin treatments on muscle flavour precursor metabolites of Jiangnan White geese.

Items	CG	HLG50	HLG100	HLG200	LG100	SEM	*p*-Value	*q*-Value
Breast muscle								
INO, mg/g	2.25 ^d^	2.38 ^c^	2.81 ^a^	2.82 ^a^	2.57 ^b^	0.06	0.0022	0.0411
IMF, %	3.15	3.26	3.35	3.26	3.36	0.02	0.0341	0.0512
TG, mg/g	3.31	3.04	2.95	3.15	3.15	0.05	0.0679	0.0750
PL, mg/g	1.07 ^d^	1.14 ^c^	1.34 ^a^	1.18 ^b^	1.32 ^a^	0.04	0.0295	0.0491
Leg muscle								
INO, mg/g	2.45 ^c^	2.60 ^b^	2.83 ^a^	2.43 ^c^	2.44 ^c^	0.05	0.0307	0.0485
IMF, %	2.87	3.08	3.10	3.11	3.08	0.04	0.0422	0.0596
TG, mg/g	3.18	3.15	2.94	3.06	3.04	0.04	0.0634	0.0750
PL, mg/g	1.08	1.17	1.34	1.34	1.21	0.05	0.0718	0.0746

INO, inosinic acid; IMF, intramuscular fat; TG, triglycerides; PL, phospholipid. ^a,b,c,d^ *p* and *q* are the unadjusted omnibus ANOVA and Benjamini–Hochberg-adjusted values, respectively. Within rows with *q* < 0.05, different lowercase superscripts describe Duncan post hoc group patterns (*p* < 0.05). CG (*n* = 6), control group; HLG50 (*n* = 6), basal diet with 50 mg/kg hydroxylated lecithin; HLG100 (*n* = 6), basal diet with 100 mg/kg hydroxylated lecithin; HLG200 (*n* = 6), basal diet with 200 mg/kg hydroxylated lecithin; LG100 (*n* = 6), basal diet with 100 mg/kg soybean lecithin; SEM, standard error of the mean.

**Table 13:** Effects of dietary lecithin treatments on muscle flavour-associated amino acid composition (g/100 g) of Jiangnan White geese.

Items	CG	HLG50	HLG100	HLG200	LG100	SEM	*p*-Value	*q*-Value
Breast muscle								
Asp	1.56 ^d^	1.48 ^e^	1.65 ^c^	1.90 ^a^	1.87 ^b^	0.021	0.0120	0.0411
Ser	0.78 ^d^	0.82 ^c^	0.90 ^a^	0.85 ^b^	0.82 ^c^	0.009	0.0200	0.0420
Glu	2.90 ^e^	2.99 ^d^	3.32 ^a^	3.13 ^c^	3.23 ^b^	0.032	0.0080	0.0436
Gly	0.84 ^e^	0.88 ^d^	1.00 ^a^	0.92 ^c^	0.95 ^b^	0.011	0.0160	0.0419
Ala	1.23 ^e^	1.37 ^d^	1.80 ^a^	1.47 ^c^	1.56 ^b^	0.042	0.0101	0.0411
Lys	0.65	0.65	0.82	0.65	0.76	0.023	0.0682	0.0749
His	1.46	1.65	1.95	1.65	1.79	0.037	0.0751	0.0420
Arg	1.00 ^e^	1.16 ^d^	1.36 ^a^	1.30 ^c^	1.33 ^b^	0.013	0.0141	
Leg muscle								
Asp	1.65 ^e^	1.85 ^c^	1.91 ^a^	1.82 ^d^	1.88 ^b^	0.020	0.0112	0.0411
Ser	0.77 ^d^	0.81 ^c^	0.85 ^b^	0.85 ^b^	0.88 ^a^	0.008	0.0232	0.0425
Glu	2.90 ^c^	2.99 ^b^	3.26 ^a^	2.99 ^b^	3.26 ^a^	0.031	0.0074	0.0423
Gly	0.85 ^e^	0.91 ^d^	1.23 ^a^	1.00 ^c^	1.16 ^b^	0.030	0.0092	0.0416
Ala	1.32 ^c^	1.33 ^c^	1.47 ^a^	1.37 ^b^	1.47 ^a^	0.012	0.0213	0.0420
Lys	0.62	0.65	0.71	0.68	0.68	0.009	0.0704	0.0750
His	1.36	1.59	1.65	1.62	1.65	0.015	0.0735	0.0746
Arg	1.26 ^d^	1.26 ^d^	1.54 ^a^	1.43 ^c^	1.46 ^b^	0.022	0.0188	0.0420

Asp, aspartic acid; Ser, serine; Glu, glutamic acid; Gly, glycine; Ala, alanine; Lys, lysine; His, histidine; Arg, arginine. ^a,b,c,d,e^ *p* and *q* are the unadjusted omnibus ANOVA and Benjamini–Hochberg-adjusted values, respectively. Within rows with *q* < 0.05, different lowercase superscripts describe Duncan post hoc group patterns (*p* < 0.05). CG (*n* = 6), control group; HLG50 (*n* = 6), basal diet with 50 mg/kg hydroxylated lecithin; HLG100 (*n* = 6), basal diet with 100 mg/kg hydroxylated lecithin; HLG200 (*n* = 6), basal diet with 200 mg/kg hydroxylated lecithin; LG100 (*n* = 6), basal diet with 100 mg/kg soybean lecithin; SEM, standard error of the mean.

**Table 14:** Effects of dietary lecithin treatments on breast muscle fatty acid composition (g/100 g) of Jiangnan White geese.

Items	CG	HLG50	HLG100	HLG200	LG100	SEM	*p*-Value	*q*-Value
C14:0	0.28	0.31	0.37	0.34	0.37	0.004	0.0211	0.0723
C16:0	24.19	24.29	20.14	23.76	24.01	1.63	0.0720	0.1082
C16:1	2.05	2.08	2.32	2.11	2.15	0.008	0.0140	0.0716
C18:0	12.96	12.03	11.23	12.15	13.21	1.01	0.0802	0.1082
C18:1 n9c	37.56	33.85	30.12	30.78	28.82	3.14	0.0772	0.0189
C18:2 n6c	14.42 ^e^	16.73 ^d^	22.72 ^a^	18.03 ^c^	20.56 ^b^	0.42	0.0061	0.0345
C18:3 n3	0.12	0.14	0.16	0.17	0.17	0.003	0.0322	0.0750
20:0	1.23	1.23	1.31	1.23	1.25	0.005	0.0411	0.0750
C20:1	0.32	0.34	0.35	0.34	0.34	0.004	0.0461	0.0756
C20:2	0.25	0.25	0.25	0.25	0.25	0.003	0.9352	0.9350
C20:3	0.14	0.15	0.15	0.15	0.17	0.003	0.0384	0.0750
C22:0	0.10 ^ab^	0.08 ^b^	0.12 ^a^	0.07 ^b^	0.08 ^b^	0.004	0.0435	0.0746
C20:4 n6	4.16 ^c^	6.24 ^b^	8.28 ^a^	8.30 ^a^	6.23 ^b^	0.32	0.0036	0.0307
C20:5	0.04	0.05	0.06	0.06	0.06	0.003	0.0916	0.1131
C24:0	0.05	0.06	0.08	0.06	0.07	0.004	0.0356	0.0756
C24:1	0.98	1.02	1.12	1.02	1.05	0.008	0.0197	0.0723
C22:6 n3	1.15	1.15	1.22	1.18	1.21	0.007	0.0241	0.0736
SFA	38.81	38	33.25	37.61	38.99	2.814	0.5856	0.6407
MUFA	40.91	37.29	33.91	34.25	32.36	2.357	0.1135	0.1368
PUFA	20.28 ^c^	24.71 ^bc^	32.84 ^a^	28.14 ^ab^	28.65 ^ab^	1.262	0.0024	0.0307
n-3 PUFA	1.31	1.34	1.44	1.41	1.44	0.076	0.7924	0.8280
n-6 PUFA	18.97 ^c^	23.54 ^bc^	31.4 ^a^	26.73 ^ab^	27.21 ^ab^	1.192	0.0012	0.0230
n-6/n-3	14.47 ^d^	17.56 ^c^	21.76 ^a^	18.94 ^b^	17.86 ^b^	0.185	<0.0001	0.0219

C14:0, Myristic acid; C16:0, Palmitic acid; C16:1, Palmitoleic acid; C18:0, Stearic acid; C18:1 n9c, Oleic acid; C18:2 n6c, Linoleic acid; C18:3 n3, α-Linolenic acid; C20:0, Arachidic acid; C20:1, cis-11-Eicosenoic acid; C20:2, cis-11,14-Eicosadienoic acid; C20:3, cis-11,14,17-Eicosatrienoic acid; C22:0, Behenic acid; C20:4 n6, Arachidonic acid; C20:5 (EPA), cis-5,8,11,14,17-Eicosapentaenoic acid; C24:0, Lignoceric acid; C24:1, Nervonic acid; C22:6 n3 (DHA), cis-4,7,10,13,16,19-Docosahexaenoic acid. SFA = C14:0 + C16:0 + C18:0 + C20:0 + C22:0 + C24:0, MUFA = C16:1 + C18:1 n9c + C20:1 + C24:1, PUFA = C18:2 n6c + C18:3 n3 + C20:2 + C20:3 + C20:4 n6 + C20:5 + C22:6 n3, n-3 PUFA = C18:3 n3 + C20:5 + C22:6 n3, n-6 PUFA = C18:2 n6c + C20:3 + C20:4 n6. ^a,b,c,d,e^ *p* and *q* are the unadjusted omnibus ANOVA and Benjamini–Hochberg-adjusted values, respectively. Within rows with *q* < 0.05, different lowercase superscripts describe Duncan post hoc group patterns (*p* < 0.05). CG (*n* = 6), control group; HLG50 (*n* = 6), basal diet with 50 mg/kg hydroxylated lecithin; HLG100 (*n* = 6), basal diet with 100 mg/kg hydroxylated lecithin; HLG200 (*n* = 6), basal diet with 200 mg/kg hydroxylated lecithin; LG100 (*n* = 6), basal diet with 100 mg/kg soybean lecithin; SEM, standard error of the mean.

**Table 15:** Effects of dietary lecithin treatments on leg muscle fatty acid composition (g/100 g) of Jiangnan White geese.

Items	CG	HLG50	HLG100	HLG200	LG100	SEM	*p*-Value	*q*-Value
C14:0	0.26	0.30	0.36	0.32	0.32	0.003	0.0171	0.0723
C16:0	23.41	23.42	20.42	23.15	22.22	1.48	0.0742	0.1082
C16:1	2.02	2.05	2.16	2.15	2.15	0.006	0.0220	0.0716
C18:0	12.21	12.33	11.40	12.62	12.92	0.97	0.0830	0.1091
C18:1 n9c	38.52	38.29	30.91	33.48	29.94	2.96	0.0794	0.1082
C18:2 n6c	16.31 ^c^	16.32 ^c^	22.33 ^a^	19.78 ^b^	22.30 ^a^	0.41	0.0055	0.0329
C18:3 n3	0.05	0.05	0.10	0.07	0.06	0.003	0.0301	0.0752
C20:0	1.05	1.05	1.17	1.11	1.13	0.004	0.0391	0.0749
C20:1	0.22	0.22	0.31	0.31	0.31	0.004	0.0442	0.0746
C20:2	0.21	0.22	0.33	0.23	0.33	0.005	0.0716	0.1079
C20:3	0.14	0.16	0.16	0.16	0.16	0.003	0.0364	0.0748
C22:0	0.03	0.03	0.06	0.03	0.03	0.003	0.0406	0.0746
C20:4 n6	3.68 ^d^	3.67 ^d^	8.12 ^a^	4.86 ^c^	6.02 ^b^	0.30	0.0044	0.0307
C20:5	0.03	0.03	0.05	0.03	0.03	0.003	0.0885	0.1124
C24:0	0.02	0.02	0.05	0.02	0.04	0.003	0.0335	0.0746
C24:1	0.91	0.91	1.01	0.97	1.01	0.007	0.0212	0.0723
C22:6 n3	0.93	0.93	1.06	1.02	1.03	0.006	0.0266	0.0747
SFA	39.14	37.11	35.32	35.31	36.97	2.91	0.902	0.9220
MUFA	39.36	41.52	38.12	35.65	35.17	3.28	0.5536	0.6204
PUFA	22.74	21.39	27.07	28.98	28.22	2.47	0.1916	0.2196
n-3 PUFA	1.07	1.01	1.12	1.17	1.12	0.09	0.7455	0.7956
n-6 PUFA	21.66	20.38	25.94	27.83	27.11	2.37	0.1781	0.2098
n-6/n-3	20.17 ^c^	20.16 ^c^	22.84 ^a^	23.88 ^b^	24.13 ^a^	0.58	0.0042	0.0306

C14:0, Myristic acid; C16:0, Palmitic acid; C16:1, Palmitoleic acid; C18:0, Stearic acid; C18:1 n9c, Oleic acid; C18:2 n6c, Linoleic acid; C18:3 n3, α-Linolenic acid; C20:0, Arachidic acid; C20:1, cis-11-Eicosenoic acid; C20:2, cis-11,14-Eicosadienoic acid; C20:3, cis-11,14,17-Eicosatrienoic acid; C22:0, Behenic acid; C20:4 n6, Arachidonic acid; C20:5 (EPA), cis-5,8,11,14,17-Eicosapentaenoic acid; C24:0, Lignoceric acid; C24:1, Nervonic acid; C22:6 n3 (DHA), cis-4,7,10,13,16,19-Docosahexaenoic acid. SFA = C14:0 + C16:0 + C18:0 + C20:0 + C22:0 + C24:0, MUFA = C16:1 + C18:1 n9c + C20:1 + C24:1, PUFA = C18:2 n6c + C18:3 n3 + C20:2 + C20:3 + C20:4 n6 + C20:5 + C22:6 n3, n-3 PUFA = C18:3 n3 + C20:5 + C22:6 n3, n-6 PUFA = C18:2 n6c + C20:3 + C20:4n6. ^a,b,c,d^ *p* and *q* are the unadjusted omnibus ANOVA and Benjamini–Hochberg-adjusted values, respectively. Within rows with *q* < 0.05, different lowercase superscripts describe Duncan post hoc group patterns (*p* < 0.05). CG (*n* = 6), control group; HLG50 (*n* = 6), basal diet with 50 mg/kg hydroxylated lecithin; HLG100 (*n* = 6), basal diet with 100 mg/kg hydroxylated lecithin; HLG200 (*n* = 6), basal diet with 200 mg/kg hydroxylated lecithin; LG100 (*n* = 6), basal diet with 100 mg/kg soybean lecithin; SEM, standard error of the mean.

## Data Availability

The data presented in this study are available on request from the corresponding author. The pen-level data generated and analysed in this study are not publicly available at present because the dataset forms part of an ongoing patent application and is subject to temporary intellectual property restrictions imposed by the authors’ institution. All treatment-level summary data supporting the conclusions are provided in the article. The underlying data will be considered for release once the relevant patent-protection procedures have been completed and disclosure has been authorised by the institution.
